# Evaluation of User Satisfaction and Trust of Review Platforms: Analysis of the Impact of Privacy and E-WOM in the Case of TripAdvisor

**DOI:** 10.3389/fpsyg.2021.750527

**Published:** 2021-09-16

**Authors:** Juan-Gabriel Martínez-Navalón, Vera Gelashvili, Alba Gómez-Ortega

**Affiliations:** Department of Business Economic, Faculty of Legal and Social Sciences, King Juan Carlos University, Madrid, Spain

**Keywords:** privacy, satisfaction, trust, E-WOM, TripAdvisor

## Abstract

Technological advances have had many advantages like an E-WOM (Electronic Word of Mouth) that has become a very important and powerful tool for users who wish to share their knowledge, experiences and emotions about a product or service. But the use of virtual platforms may affect the privacy of users data. This present study has a twofold objective: first check if the privacy of users when using TripAdvisor, the world’s largest travel platform, has an impact on their satisfaction and trust on the platform. Secondly, the relationship between E-WOM and the variables users trust and satisfaction when using TripAdvisor is examined. In order to achieve the objectives set out, the sample of 390 persons was analyzed. The PLS-SEM method has been used to process the data and test the hypotheses. The results of the analysis have shown that there is a positive and direct relationship between TripAdvisor users’ privacy and satisfaction. The direct and positive relationship between users’ E-WOM and their degree of satisfaction and trust toward the platform has also been confirmed. This study makes a significant contribution to the academic literature on the variables studied, as previous studies presented different results.

## Introduction

Technological development and its application in social networks have had a significant impact on the way in which consumers exchange their experiences and opinions. Authors such as Bigné et al. (2013) note the influence that the exchange of online experiences has on the attitude toward the brand and the brand’s influence on purchase intention. According to Alonso (2016), digital tools create groups of belonging that condition behavior, as they create widely accepted trends of opinion. Consumers are the main promoters of brands, due to the frequency in the use of social networks, they have the power to promote or discredit a company in a matter of seconds, even at an international level (Apolo et al., 2015). This is why companies should take into account digital marketing strategies (Saura, 2020).

According to Sánchez and Arroyo-Cañada (2016), there are important differences between the traditional shopping process and online shopping. One of them is the degree of technological interaction (Agudo-Peregrina et al., 2014). The use of digital platforms for reviews and opinions is one of the most important tools in terms of consumer interaction. The emergence of these platforms has significantly reduced transaction costs, which explains the enormous prominence they have acquired in recent years (Belleflamme and Peitz, 2018). These authors argue that reviews and feedback are an almost consubstantial element of digital platforms. During the purchasing process, consumers seek evaluations and recommendations about the attributes of a product or service, and to do so, they use various sources of information, including these platforms (Pérez-Aranda et al., 2017). As these authors point out, the final purchase decision of users is closely linked to E-WOM, so it is necessary to analyze how organizations, and especially hotels, manage the evaluations shared on line by users. Gil et al. (2017) state that the tourism sector, and specifically the hotel sector, is particularly vulnerable to the opinions generated by travelers (Buhalis and Law, 2008) and is clearly affected online by the so-called E-WOM.

On the other hand, the technological development and exponential growth of digital platforms, mentioned above, makes it essential to analyze user privacy and its impact on the management of these tools. This problem, applied to social networks, is highly debated (Kayes and Iamnitchi, 2017). However, we cannot forget the importance that privacy can have for a platform where users can express their opinion about a service received. Understanding that the opinion may be conditioned by the continuity of the service in the future and by the relationship with the company that offers it. For this reason privacy management of user information is becoming increasingly complex (Trepte, 2020).

Given the above, this study has a twofold objective. On the one hand, to analyze how privacy affects user satisfaction and trust, when using review platforms. On the other hand, whether E-WOM has an impact on these variables. To carry out the study, TripAdvisor has been selected as one of the most important digital platforms (Balagué et al., 2016; Yoo et al., 2016; Gil et al., 2017; Mariottini and Toribio, 2017).

The data were collected through an online questionnaire that collected users’ opinions about TripAdvisor. After the current introduction, the theoretical framework is developed, setting the studied variables: privacy, E-WOM, satisfaction and trust. Subsequently, the 5 hypotheses to be tested are presented and justified, followed by an explanation of the research model. Next, the analysis of the data collected will be presented, ending with a discussion of the results and the main conclusions of the study.

The main results show that there is a positive and direct relationship between TripAdvisor users’ privacy and satisfaction. Meanwhile, the hypothesis that analyzed the relationship between TripAdvisor users’ privacy and trust has been rejected. The direct and positive relationship between users’ E-WOM and their degree of satisfaction and trust toward the platform has also been confirmed.

The interest of this paper lies in finding managerial implications for Tripadvisor’s future strategies. In the literature review, no previous studies have been found that analyze privacy and E-WOM hand in hand, in order to know their impact on user satisfaction and trust.

## Theoretical Framework

### Privacy

Technological evolution has changed life for all people, achieving greater efficiency, products and services that were not previously available to users, thereby improving conditions and the quality of life in general (Funk et al., 2018; Chen et al., 2021). People use electronic tools on a daily basis, which in turn provide different Applications (hereafter Apps) that make it possible to access various services and products simply by registering (Sturm et al., 2018; Tao and Edmunds, 2018). For example, using Apps to move around the city (Uber, Cabify, car sharing Apps) records information about where a person moves, shopping Apps (supermarkets, clothes shops, make-up shops, etc.) store information about a person’s tastes and preferences, or simply searching for a term in a search engine stores the searched information. On the basis of these searches and purchases a database is created for a specific person and all adds or offers then take into account the previously collected information (Goldenberg et al., 2012; Fainmesser et al., 2019). Because of this, companies have the possibility to design specific offers for their customers (Gelashvili et al., 2021), but the big question is whether this affects or not user’s privacy and security on digital platforms (Saura et al., 2021a,b) since, actually more and more personal information is being processed, shared or disseminated by companies (Ribeiro-Navarrete et al., 2021).

Therefore, one of the drawbacks of this technological advance is a lack of privacy, in particular digital privacy (Munuera, 2017). Privacy is understood as *a matter of controlling one’s own data, including information disclosure* (Sarikakis and Winter, 2017). Study elaborated by Sarathy and Robertson (2003) indicates that individualized information based on users’ activities on online platforms allows companies to offer personalized marketing, but this may cause an alarm about the private data of individuals.

The problem of data privacy is particularly visible in social networks (Kayes and Iamnitchi, 2017). Increasingly, users face a lack of control over their information on social networks as they cannot control what companies or other users do with the information they share online. This is why the control of individual users’ information has become more difficult (Trepte, 2020). As stated by Beigi and Liu (2018), the daily use of social networks by users has generated an enormous amount of user-generated data that is used by researchers and service providers being an opportunity for them, but that in turn creates a risk of exposing individuals’ privacy. According to Fainmesser et al. (2019) in recent years, there has been growing concern about the privacy and security of users’ data, for this reason governments have designed and implemented new laws and regulations that do not provide 100% protection but are partially effective at best.

### E-WOM

Nowadays, the accessibility of online platforms has enabled users to change their opinions about products and services. On the basis of these opinions, many customers decide whether or not to consume a service and purchase a product (Jiménez-Castillo and Sánchez-Fernández, 2019; Nuseir, 2019). This method of exchange or expression of consumer-generated opinions on the Internet is called E-WOM. Usually these opinions are addressed to other users. One of the first definitions of E-WOM (Hennig-Thurau et al., 2004) states that E-WOM is *any positive or negative statement made by potential, actual or former customers about a product or company, which is made available to a multitude of people and institutions via the Internet*. E-WOM has a major impact on both businesses and their consumers (López and Sicilia, 2014). Through social networks and electronic channels consumers can express their opinion which is visible to the rest of the users, if these comments are negative other consumers will avoid buying or acquiring those products and services which will generate a decrease in sales for the companies (Sa’ait et al., 2016; Li et al., 2019). In addition to this, if companies will not take into account the importance of E-WOM for businesses, there will be a high probability with customer satisfaction, loyalty and trust (Rialti et al., 2017; Duarte et al., 2018; Mishra and Singh, 2019; Tran and Strutton, 2020).

According to Reyes-Menendez et al. (2019) there are different social network sites or platforms like Facebook, Instagram, Twitter, Amazon, Expedia or TripAdvisor that can be useful to inform about the features or benefits of products and services. With a special focus on TripAdvisor, it can be considered as one of the most important E-WOM platforms in the tourism sector. The comments and opinions generated by users generate trust in other users, for this reason it is necessary to manage correctly the content on platforms since there may be some false opinions that can affect the reputation of companies.

But E-WOM is not only in the tourism sector that has an importance. Study elaborated in the banking sector have shown that E-WOM is positively related to loyalty, trust and purchase intention (Guping et al., 2021; Zhang et al., 2021). Another study elaborated by Tjhin and Aini (2019), has found that E-WOM has a significant effect on purchase decision and consumer trust in clothing industry. This means that in almost all industries E-WOM has an important significance. For this companies need to be aware of the huge impact E-WOM has on consumers and to avoid problems they need to pay special attention to customer satisfaction and complaints (López and Sicilia, 2014).

### Satisfaction

Nowadays user satisfaction plays an important role for the performance and growth of the companies (Eklof et al., 2020; Otto et al., 2020). User satisfaction is defined as the users’ opinion of the service or products received compared to the service or products expected (Jani and Han, 2011). This means that if the customer has expectations of receiving a product or service of a certain quality and when they receive these products and services in line with expectations, satisfaction is generated. Therefore, through customer satisfaction companies can know the tastes and preferences of their customers (Alsini, 2017).

Through satisfaction it is possible to study the trust and loyalty generated by users toward the company or products. According to Gelashvili et al. (2021) when using mobile Apps for restaurant reservation has a positive and direct impact on users’ trust in restaurants. Likewise, researches carried out on tourism companies have shown that when the user of these companies is satisfied with the services, trust is generated (Lai, 2014; Martínez-Navalón et al., 2019). In addition to trust, another variable that has been studied along with satisfaction by several studies is loyalty (El-Adly, 2019; Thamrin et al., 2020). These studies have shown that user satisfaction is positively related to user loyalty toward the brand. In other words, if satisfaction is generated toward a product or service, this positively affects the loyalty of customers, who will continue to buy the same products and services. Therefore, we can say that these two variables are important for companies to be taken into account. Apart from these two variables, the direct and positive relationship of customer satisfaction with the variables like continuance intention or service quality among others has been studied (Chumpitaz and Paparoidamis, 2007; Liang et al., 2018; Hossain et al., 2019).

In short, user satisfaction is a key factor for companies, because a satisfied customer is a customer who intends to repurchase, trusts the brand and is loyal to the company’s products and services. This implies a long term relationship between the customer and the company.

### Trust

The variable trust is one of the most important variables in analyzing customer behavior and has been the subject of research over the decades (Swan et al., 1985; Komiak and Benbasat, 2004; Leninkumar, 2017; Al-Khalaf and Choe, 2020). According to McKnight and Chervany (2001) it is not easy to define the term trust, as most definitions are confusing and for this reason some researchers have chosen not to go further in defining the concept. This may be because each discipline defines and analyses trust from its own perspective. In social sciences customer trust is referred to *as the willingness of the consumer to rely on a service brand because he or she has confidence in the reliability and integrity of that brand* (Zhang and Bloemer, 2008). In other words, trust could be defined as the users’ confidence or faith in the brand’s products and services. According to Martínez-Navalón et al. (2019) a customer who has trust in the brand’s products and services could help the company improve its profitability and achieve other business goals. Another advantage for companies is that the trust variable creates a stable and collaborative user-company relationship (Bilgihan and Bujisic, 2015). As we can see, it is essential for companies to create the trust of their users toward their products and services, which will help to improve their economic and financial situation as well as competitiveness in the market.

Study carried out by Aurifeille et al. (2009), on the measurement of trust indicates that it is not easy to measure thisvariable since there are several studies that analyze customer trust based on various dimensions, among them it can be seen the following dimensions: reliability, credibility, honesty, benevolence, expectation or confidence among others. Lassala et al. (2010) to measure customer trust, uses the following three dimensions: competence, benevolence and honesty, where honesty is defined as the belief that the other party will fulfill the promises and obligations undertaken. Competence refers to the professionalization of companies in terms of promised quality, know-how and ability to do their job well. And finally, the dimension of benevolence refers to the belief that the other party is interested in achieving joint benefits and will make decisions that will be beneficial to both parties. Several recent studies (Martínez-Navalón et al., 2019; Gelashvili et al., 2021) have supported the use of these three dimensions to measure customer trust toward a company’s products or services.

## Hypotheses and Research Model

One of the industries where E-WOM is most important is the tourism sector (Nieto-García et al., 2017; Kanje et al., 2020). As usual, people before making a trip always look at the content generated on websites about different tourist destinations, quality of hotels or the hospitality industry in general. In case of a set of negative or unfavorable comments on sites such as TripAdvisor, Google Maps, Airbnb or Booking among others may be detrimental to future customers. All these Apps allow consumers to express their opinion about a service or product in the tourism sector (Wu et al., 2013; Reyes-Menendez et al., 2019; Boukherouk et al., 2020). But undoubtedly the most used by consumers or future consumers is TripAdvisor (Valdivia et al., 2017; Reyes-Menendez et al., 2019).

TripAdvisor was founded in the United States in 2000 and is currently the most popular platform in the tourism sector where users can find reviews and information about tourist destinations, restaurants and other tourist attractions (Miguéns et al., 2008; O’connor, 2008; Valdivia et al., 2017). That’s means that the users can find the information they need to make their travel decisions based on other users review. The popularity and evolution of this platform is directly related to E-WOM as the relationship between users is through the internet and opinions (Fili and Križaj, 2017; Reyes-Menendez et al., 2019). But to post a review on TripAdvisor to generate E-WOM content about a tourist destination, restaurant or tourist experience requires registration, which is related to the provision of personal data that may affect users’ privacy.

Privacy for the user of a service, in the digital framework, is an issue of transcendental relevance and there are numerous recent research studies that analyze this variable and its impact on the user (Chung et al., 2021; Lax et al., 2021; Pilton et al., 2021; Saura et al., 2021c; Wu et al., 2021). This has led competent authorities to develop specific regulations that allow users to manage their privacy preferences when they log on to a platform (Tamburri, 2020; Curry, 2021). Within the European Union (European Commission, 2016), the General Data Protection Regulation 2016/679 was developed in 2018 (hereafter, GDPR). According to Politou et al. (2018), *the GDPR encompasses some new data protection principles for giving control back to individuals over their personal data such as the right to object to profiling, the right to data portability, and the obligation for data protection impact assessments*. This raises the question of whether or not user privacy can have an impact on the satisfaction of a service received. Some authors have shown that privacy risk leads to a reduction in user satisfaction (Cheng and Jiang, 2020). However, Kim et al. (2012) in their work claim that there is no such relationship. Therefore, taking into account the importance of privacy as a variable and the difference in the results of the aforementioned research, the first hypothesis to be tested is the following:

**H_1_** The privacy of users when using TripAdvisor has a direct and positive impact on their satisfaction on the platform.

Other authors have also linked privacy to user trust. Mutimukwe et al. (2020), claim that perceptions of privacy risk influence user trust and behavior. Aïmeur et al. (2016), conduct an experimental study to compare user trust under two different privacy policies. These authors conclude that changing the appearance of privacy policies makes online services appear more trustworthy to the user. For its part, Voloch et al. (2021), propose a model where user privacy policy is conditioned by three significant aspects, trust, role-based access control and information flow. Due to the relationship shown in different studies between privacy and trust, the second hypothesis to be analyzed is the following:

**H_2_** The privacy of users when using TripAdvisor has a direct and positive impact on their trust on the platform.

Emotions and sensations determine consumer behavior in many cases. According to Heyes and Kapur (2012), the growth of E-WOM affects quality choices. A customer experiencing poor quality may decide to change supplier. The E-WOM phenomenon and its impact on consumer decisions has been analyzed on numerous occasions (Goyette et al., 2012; López and Sicilia, 2014; Yoo et al., 2016; Krishna and Kim, 2020). However, the existence of so-called “bots” can lead to some manipulation of the opinion of a service (Cheng et al., 2020). These bots consist of automated processes that generate mass reviews, positive or negative, created for a specific purpose (Orabi et al., 2020). All of the above makes it essential to understand how the processes of opinion transmission between consumers are generated and the extent to which they affect consumer decisions (Wang et al., 2018; Ismagilova et al., 2019). In this sense, there are different studies that relate E-WOM to user satisfaction (Aakash and Aggarwal, 2019; Serra-Cantallops et al., 2020; Tandon et al., 2020). The analysis of this relationship in the specific field of review platforms leads to the third hypothesis:

**H_3_** The E-WOM on TripAdvisor has a direct and positive impact on the satisfaction of users of the platform.

As a continuation of the previous hypothesis, studies have also been identified that relate E-WOM to the trust generated in the user (Ladhari and Michaud, 2015; Reimer and Benkenstein, 2016; Bhandari and Rodgers, 2017). Therefore, we propose the fourth hypothesis to be tested in this study:

**H_4_** The E-WOM on TripAdvisor has a direct and positive impact on the trust of users of the platform.

Numerous studies have analyzed the relationship between user satisfaction and user trust in different application areas. Some authors have identified a direct relationship between the two and the quality of service (Dwyer et al., 1987; Crosby et al., 1990; Shamdasani and Balakrishnan, 2000; Henning-Thurau et al., 2001; Chumpitaz and Paparoidamis, 2007). Other authors consider that both variables have a significant impact on consumer loyalty (Hossain et al., 2019; Thamrin et al., 2020). More recent studies such as Martínez-Navalón et al. (2019), show how in tourism companies, user satisfaction on social networks influences trust in these companies. Lai (2014) demonstrated the relationship between satisfaction with a travel agency and improved trust in the travel agency. Another study elaborated by Liang et al. (2018), explores the relationship between satisfaction, trust and repurchase intention. Gelashvili et al. (2021) demonstrated that the satisfaction of users who make restaurant reservations via a mobile Apps has a direct impact on trust in those restaurants. Based on the above, we consider it interesting to formulate the following hypothesis, to study the relationship between satisfaction and trust as applied to a travel service review and rating platform:

**H_5_** The satisfaction of users when using TripAdvisor has a direct and positive impact on their trust on the platform.

**FIGURE 1:**
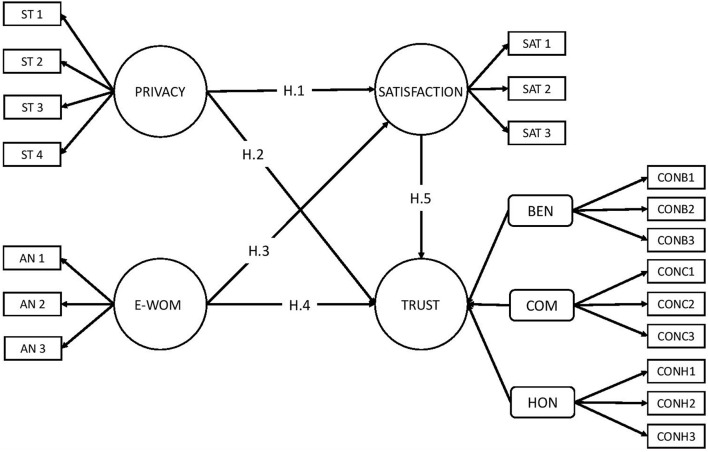
Proposed research model. Source: own elaboration.

Taking into account all of the hypotheses set out above, the following research model is proposed (Figure 1):

In consideration of the review of the literature on the study’s main variables, it is expected that all hypotheses will be accepted.

## Data Analysis

### Data Collection

The objective of this study was to study users who use the TripAdvisor website with the aim of finding managerial implications for the company’s future strategy approach. Therefore, it analyses how privacy and E-WOM affect the satisfaction and trust of users of this review website in tourism and hospitality.

In order to carry out this study, an online questionnaire data source was used. This questionnaire has had a self-administered format that has collected the opinion of users of this Apps living in central Spain. Specifically in the regions of Castilla-La Mancha and Madrid. Focusing on localities such as Albacete, Chinchilla de Montearagón, Guadalajara, Cuenca, Madrid, Chinchón, Arroyomolinos, Móstoles, Fuenlabrada, etc. This sample is motivated by the intention of finding out the opinion of the population in the interior of Spain. Data collection took place during the months of May and June 2021. In order to have a greater veracity of the data, the IP of the users was checked, avoiding that the same user fills in the form twice.

The data collection process was divided into two stages. In the first stage, a sample of 50 questionnaires was collected in order to carry out a pre-test. Once it was verified that the measurement scale was well done, the second part was developed, which was the collection of the majority of data, where 400 questionnaires were obtained. Of these sample, 390 were valid.

The questionnaire used in this study was divided into two parts. In the first part, questions were asked to classify the users. For this classification, questions were asked about gender, employment status, age and education. For the second part of the questionnaire, a measurement scale was used, based on the bibliographical analysis carried out in this study. It analyses the variables privacy, E-WOM, satisfaction and trust. It should be noted that the trust variable is a multidimensional variable, so an analysis by dimensions (Benevolence, Honesty and Competence) had to be carried out. A total of 19 questions were asked to measure these variables.

The type of questions used were Likert scale questions from 0 to 6 (1 = “strongly disagree”; 6 = “strongly agree”). The choice of these types of questions is motivated by the fact that they are able to analyze the degree of sensitivity of each user and are the most commonly used type of question in social science studies (Fernández-Fernández et al., 2021).

### Method of Data Analysis

In order to carry out the validation of the measurement scale and the validation of the hypotheses put forward in the model, a structural equation analysis based on variances has been carried out (Liengaard et al., 2021). The PLS-SEM method through the SmartPLS software has been used to process the data and test the hypotheses (Del-Castillo-Feito et al., 2020).

This is a multivariate analysis method whose main purpose is the prediction of dependent variables by estimating robust models. This program allows to analyze and determine the estimation of the measurement model and structural model taking into account their dependent variables. It also allows for the calculation and quantification of the size of both indirect and direct effects that some of the variables in this model have on others (Cachón-Rodríguez et al., 2021). The method has the advantage of deciding whether or not to impose the direction of the hypotheses and is considered to be the most reliable and advisable according to Hair et al. (2019).

The composition of the proposed model, which includes reflective and formative variables, makes this technique and software optimal for the analysis proposed in the study (Martínez-Navalón et al., 2020).

## Analysis of the Results

Before starting the analysis of the proposed model in this study, the structure to be developed for this analysis must be taken into account. The first thing to do is to validate the measurement scale in order to be able to carry out the analysis of the model. The validation of the measurement scale of this model is a two-step validation, due to the fact that there is a multidimensional variable (trust) in this model. In order to validate a model with a multidimensional variable, all the items that make up the dimensions must first be analyzed and then grouped together to form an item for each dimension. The first step to be carried out is called “validation of the measurement scale of the first order model” and the second step is called “validation of the measurement scale of the second order model” (Hair et al., 2019).

During the validation analysis of both the first and second order measurement scale, items that do not meet the requirements for the validation of the measurement scale will be eliminated, and then proceed with the measurement of the model (Gelashvili et al., 2021).

### Analysis of the Survey Population

Before starting with the analysis of the measurement scale and the analysis of the model, the classificatory data analysis of the sample obtained from the questionnaires is carried out. This analysis shows the data collected in the first part of the questionnaire and shows the characteristics of the sample.

Gender, employment status and age are studied in this analysis. This helps to have knowledge of the sample obtained. This makes it possible to specify to which levels of the population or styles of the population the results obtained in this work can be applied. The sample consists of 390 questionnaires (400 collected and 390 valid) (Table 1).

**TABLE 1:** Sample characteristics (*n* = 390).

Classification variable	Variable	Frequency	Percentage
Gender	Male	167	42.82%
	Female	223	57.18%
Age	<20	36	9.23%
	21–35	255	65.38%
	36–55	54	13.85%
	56–65	39	10.00%
	>65	6	1.54%
Employment status	Unemployed	30	7.69%
	Self-employed	24	6.15%
	Employed	185	47.44%
	Student	133	34.10%
	Retired	18	4.62%

*Source: own elaboration.*

The descriptive analysis visible in Table 1 shows that the composition of the sample is mostly female. The female gender occupies 56.92% of the individuals surveyed, compared to 43.07% of the male gender. With regard to the age composition of the sample, it can be seen that the majority of the sample is made up of individuals aged between 21 and 35, followed by individuals aged between 36 and 55. Marginally the third group of individuals surveyed are in the age range 56–65 years. Finally, with regard to the employment status of the individuals, it can be seen that the majority of the sample is made up of employees (47.43%), followed by 34.41% of students.

### Measurement Model

To carry out the analysis of the proposed model, a validation analysis of the measurement scales must be carried out, as specified above, in order to subsequently carry out the analysis of the proposed relationships (Nitzl et al., 2016). Firstly, and given that this is an analysis with multidimensional variables, we begin by validating the measurement scale of the first-order model and, once the dimensions of the trust variable have been validated, we proceed to the validation of the complete measurement scale, known as the second-order scale (Hair et al., 2018). In the first-order model we find that all items have a reflective character, while in the second-order model we observe that the variables privacy, E-WOM and satisfaction have a reflective character and that the variable trust has a formative character (Martínez-Navalón et al., 2020).

When carrying out the validation of the measurement scale, different criteria are applied for the reflective and formative items. In the case of the reflective items, an analysis of individual reliability, composite reliability, convergent validity and discriminant validity is carried out. These analyses can be found in Tables 2, 3 of the paper. In the case of the formative variables, the collinearity of the indicators is assessed through the analysis of the variance inflation factor (VIF) and their weights (Rodríguez et al., 2019).

**TABLE 2:** Measurement items first order.

Constructs	Items	Correlation loading	CA	CR	rho_A	AVE
Satisfaction	ST-1 I am satisfied with the reviews on TripAdvisor.	0.919***	0.805	0.911	0.807	0.672
	ST-2 TripAdvisor always meets my expectations.	0.911***				
Privacidad	PRIV-1 TripAdvisor explains and respects the privacy policy.	0.892***	0.883	0.918	0.918	0.737
	PRIV-2 I don’t mind giving my details to TripAdvisor because they are protected.	0.802***				
	PRIV-3 I have peace of mind when browsing TripAdvisor because I know that my information is protected.	0.923***				
	PRIV-4 When I write my reviews on TripAdvisor it does not show personal information.	0,809***				
E-WOM	EW-1 Before I travel I check the opinions of other tourists on TripAdvisor.	0.865***	0.883	0.89	0.890	0.672
	EW-2 TripAdvisor has enough references (hotels, restaurants, monuments) with opinions of other tourists.	0.818***				
	EW-3 TripAdvisor allows me to choose the best tourist destination.	0.883***				
	EW-4 TripAdvisor reviews always get it right.	0.700***				
Trust honesty	CONH-2 TripAdvisor is transparent about the reviews it stores.	0.905***	0.858	0.914	0.914	0.779
	CONH-3 TripAdvisor is ethically and transparently managed.	0.882***				
	CONH-4 I can trust TripAdvisor.	0.860***				
Trust benevolence	CONB-2 TripAdvisor develops actions taking into account that they will have an impact on its users.	0.874***	0.700	0.863	0.863	0.758
	CONB-3 TripAdvisor takes into account its stakeholders (users and destinations, restaurants, hotels, etc., about which they give their opinions) so as not to harm them.	0.868***				
Trust competence	CONC-2 TripAdvisor shows the necessary capacity to be able to carry out its work.	0.895***	0.782	0.902	0.902	0.821
	CONC-3 TripAdvisor performs competently as a review site.	0.917***				

*CA, Cronbach’s alpha; CR, Composite reliability; rho_A, Dijkstra-Henseler indicator; AVE, Average Variance Extracted.*

***p < 0.001.

*Source: own elaboration.*

**TABLE 3:** Measurement of the fist-order model (discriminant validity).

Heterotrait-Monotrait Ratio (HTMT)						
	E-WOM	Privacy	Satisfaction	Trust benevolence	Trust competence	Trust honesty
**E-WOM**						
Privacy	0.078					
Satisfaction	0.855	0.075				
Trust-benevolence	0.856	0.12	0.757			
Trust-competence	0.869	0.082	0.873	0.864		
Trust-honesty	0.797	0.1	0.83	0.822	0.754	

*Source: own elaboration.*

First, we proceed to the individual reliability analysis of the study. In this analysis, the item loadings (λ) are analyzed. These charges according to the criterion of Carmines and Zeller (1979) should be above 0.707. In this study, all items of the proposed first-order model exceed this cut-off threshold. Secondly, the validation analysis of the composite reliability scale is carried out, where the Cronbach’s alpha analysis is taken into account (Martínez-Navalón et al., 2019). The criterion used is that of Nunnally and Bernstein (1994) which sets an item validation cut-off at 0.7. Here too, all of the items raised pass this cut-off, as they all exceed 0.7. To analyze composite reliability, the Dijkstra-Henseler analysis (rho_A) is used, a more up-to-date criterion that is more robust in its analysis (Dijkstra and Henseler, 2015). Becoming the only real measure of reliability (Hair et al., 2019). The cut-off score is 0.7. In both composite reliability criteria all the items tested pass the analysis.

The convergent validation analysis uses the average variance extracted (AVE) in which we study how much of the variance of a variable is generated by its indicators (Martínez-Navalón et al., 2020). It is analyzed to ensure that the proposed indicators account for at least 50% of the variance of the underlying variable. The cut-off rate is 0.5 (Rodríguez et al., 2019). In this analysis, all of the items in this study exceed the cut-off threshold of 0.5 (Table 2).

Finally, for the validation of the measurement scale of the first-order variables, discriminant validity analysis is carried out. This study is carried out using Heterotrait-Monotrait analysis (HTMT). This criterion is the most current and allows the study to be more robust (Dijkstra and Henseler, 2015). It takes into account that the amount of variance of a variable captured from the indicators must be greater than the variance it shares with other variables (Ghasemy et al., 2021). Values in the study must be less than 0.90 (Henseler et al., 2015). Having carried out the analysis in the proposed model, the items SAT-3, CONH-1, CONB-1, CONC-2 are eliminated as they do not comply with the proposed criteria (Table 3).

Once the validation of the measurement scale of the first-order model has been completed, the validated items of the multidimensional variable (trust) are grouped together. The validation analysis of the measurement scale is carried out again, but in this case of the second-order model. In this second analysis, the reflective variables (privacy, E-WOM and satisfaction) meet all the criteria previously analyzed, and the scale for measuring these variables is considered to be validated. Subsequently, the validation of the formative variable (trust) is carried out, as the measurement scale of the formative variables is validated with different criteria. First, the weights are analyzed to see if they are significant (Gelashvili et al., 2021). All three items of the trust variable in the second order are significant. Once the weighting criterion has been overcome, the collinearity study of the items is carried out. This analysis is performed using the VIF (Hair et al., 2018). The chosen cut-off threshold is the one advocated by Hair et al. (2019) where it is advised that VIFs should be close to value 3, preferably with a lower value. Applying this criterion, the three items of the trust variable are retained (Table 4).

**TABLE 4:** Measurement of the second-order model.

Constructs	Dimensions	Correlation (weights)	VIF
Trust	Honesty	0.454***	1.914
	Benevolence	0.162***	1.956
	Competence	0.523***	1.924

*VIF, Variance Inflation Factor.*

***p < 0.001.

*Source: own elaboration.*

The measurement analysis is carried out, where it is analyzed whether the hypotheses are fulfilled or rejected.

### Structural Model Analysis

In order to carry out the structural analysis of the model previously proposed, it is necessary to analyze whether there is multicollinearity between the antecedent variables and the endogenous variables. For this purpose, structural variance inflation is analyzed using the (VIF) criterion, where the structural model must have values less than 3 according to Hair et al. (2019). In this case, the structural model meets this criterion as there is no structural multicollinearity since all values are below 2. The goodness-of-fit index of the model must also be measured beforehand. For a model to be considered a good fit, the SRMR index must be less than 0.08 (Hu and Bentler, 1998). The fit index of this model is 0.06 and therefore meets this criterion.

Once the previous analyses have been carried out, a bootstrapping analysis of 50,000 samples is applied, which allows us to see the algebraic sign, the magnitude and the significance of the hypotheses put forward. In the analysis of the algebraic sign, it can be seen that all the hypotheses have the same value as the hypothesis that has been put forward. The Student’s *t*-test and significance analysis shows that hypotheses 1, 3, 4, and 5 meet the criteria for both analyses, while hypothesis 2 does not meet the criteria. Therefore, the hypothesis H.2 (Privacy → Trust) is rejected and H.1 (Privacy → Satisfaction), H.3 (E-WOM → Satisfaction), H.4 (E-WOM → Trust), H.5(Satisfaction → Trust) are accepted (Table 5).

**TABLE 5:** Comparison of hypotheses.

	Path coeff (*ℬ*)	Statistics T (*ℬ*/STDEV)	f^2^
H1. Privacy → Satisfaction	0.1*	2.43	0.02
H3. E-WOM → Satisfaction	0.71***	24.76	0.99
H4. E-WOM → Trust	0.47***	10.65	0.37
H5. Satisfaction → Trust	0.441***	9.482	0.33

*R^2^: Trust = 0.704; Satisfaction = 0.5.*

*R^2^ tight: Trust = 0.701; Satisfaction = 0.498.*

*Q^2^: Trust = 0.511 Q^2^: Satisfaction = 0.414.*

*p < 0.05; **p < 0.01; ***p < 0.001.

*Source: own elaboration.*

Once the hypotheses have been accepted and rejected, the analysis of explained variance (R^2^) and effect size (F^2^) is carried out to measure the predictive relevance of the model (Aldás-Manzano, 2014; Hair et al., 2019). The R^2^ for trust and the R^2^ for satisfaction show positive values. In the case of trust it has high values according to Chin (1998) and in the case of satisfaction it has average values. This indicates that the predictor variables have a high predictive power. F^2^ shows how an exogenous variable contributes to explain another endogenous variable. The E-WOM variable exerts a large explanatory effect on the satisfaction and trust variables. In the case of the privacy variable, it has a low explanatory effect on satisfaction. Finally, we measure the predictive relevance of the model by (Q2), obtaining a high predictive relevance (Geisser, 1975; Martínez-Navalón et al., 2019).

**FIGURE 2:**
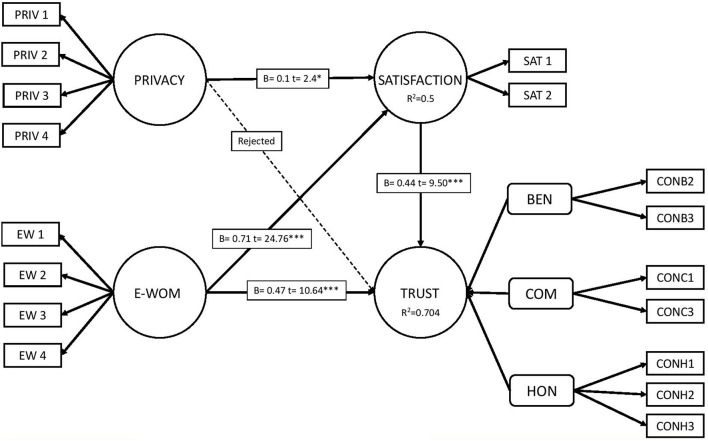
Final model. Source: own elaboration. ^∗∗∗^*p* < 0.001.

Table 5 and Figure 2 show the results obtained in the study.

## Conclusion

TripAdvisor is one of the largest platforms for reviews in the tourism sector where users express their opinions about the services they receive. These opinions can have an impact on the decision of other users, therefore the impact of this platform for tourism and hospitality companies as well as for its users is very important. Therefore, the two main objectives of this study were to analyze if the privacy of users when using TripAdvisor, has an impact on their satisfaction and trust on the platform and examine the relationship between E-WOM and users trust and satisfaction when using TripAdvisor.

The first block of analysis studied the relationship between TripAdvisor users’ perceptions of safety and the trust and satisfaction generated. The results have shown that user privacy on the TripAdvisor platform has a direct and positive relationship with user satisfaction. Therefore H1 is accepted. This result is an important contribution to the academic literature since several studies had denied positive relationship between these two variables (Cheng and Jiang, 2020) and others had confirmed the non-existence of this relationship (Kim et al., 2012). With regard to relation between users privacy and trust results have shown that there is no relationship between these two variables. On this basis H2 was rejected. This means that users’ privacy does not condition their trust on the platform.

The second block analyzed the relationship between E-WOM and TripAdvisor user satisfaction and trust. The results have shown that E-WOM has a direct and positive relationship with the satisfaction and trust of the platform’s users. On this basis the H3 and H4 have been accepted. This result is in line with previous researches (Bhandari and Rodgers, 2017; Tandon et al., 2020) and contributes to the academic literature for one of the world’s largest platform of opinion. Finally, the relationship between the variables satisfaction and trust of users of the TripAdvisor platform has been analyzed. The result showed a direct and positive relationship between these two variables. This means that if a user of this platform is satisfied with the service, this generates a higher degree of trust toward the platform. Therefore the H5 has been validated. The relationship between satisfaction and trust has been identified by other authors (Lai, 2014; Martínez-Navalón et al., 2019) but in the tourism sector, not specifically for the TripAdvisor platform.

Taking into account the results of the study, this research has theoretical and practical implications. In case of theoretical implications the importance of the study in the academic literature can be highlighted. The literature on some of the points analyzed in this research has not been uniform and clear. Therefore, this study contributes to the academic literature on the variables analyzed (privacy, E-WOM, satisfaction, trust). In case of practical implications we can highlight the importance of E-WOM for user satisfaction and trust, therefore the managers of TripAdvisor or other similar digital platforms should take into account the importance of the positive and direct relationship between E-WOM and satisfaction and trust of the users.

The present research has several limitations. First, only one review platform is analyzed, it would be interesting to compare whether the result would be the same for users of other Apps such as Google maps or Booking. Second, another methodology could be used to reinforce the results obtained. Thirdly, the sample could be increased and differentiated by gender as many recent studies point to the importance of this variable. Taking into account the limitations outlined in this study, future research lines could focus on overcoming these gaps.

## Data Availability Statement

The raw data supporting the conclusions of this article will be made available by the authors, without undue reservation.

## Author Contributions

All authors listed have made a substantial, direct and intellectual contribution to the work, and approved it for publication.

## Conflict of Interest

The authors declare that the research was conducted in the absence of any commercial or financial relationships that could be construed as a potential conflict of interest.

## Publisher’s Note

All claims expressed in this article are solely those of the authors and do not necessarily represent those of their affiliated organizations, or those of the publisher, the editors and the reviewers. Any product that may be evaluated in this article, or claim that may be made by its manufacturer, is not guaranteed or endorsed by the publisher.
